# Development of an E-Nose System for the Early Diagnosis of Sepsis During Mechanical Ventilation: A Porcine Feasibility Study

**DOI:** 10.3390/s25113343

**Published:** 2025-05-26

**Authors:** Stefano Robbiani, Louwrina H. te Nijenhuis, Patricia A. C. Specht, Emanuele Zanni, Carmen Bax, Egbert G. Mik, Floor A. Harms, Willem van Weteringen, Laura Capelli, Raffaele L. Dellacà

**Affiliations:** 1 Department of Electronics, Information, and Bioengineering, Politecnico di Milano, 20133 Milano, Italy; stefano.robbiani@polimi.it (S.R.); emanuele.zanni@polimi.it (E.Z.); raffaele.dellaca@polimi.it (R.L.D.); 2 Department of Neonatal and Pediatric Intensive Care, Division of Neonatology, Erasmus MC Sophia Children’s Hospital, University Medical Center Rotterdam, 3015 GD Rotterdam, The Netherlands; l.tenijenhuis@erasmusmc.nl (L.H.t.N.); w.vanweteringen@erasmusmc.nl (W.v.W.); 3 Laboratory of Experimental Anesthesiology, Department of Anesthesiology, Erasmus MC, University Medical Center Rotterdam, 3015 GD Rotterdam, The Netherlands; p.specht@erasmusmc.nl (P.A.C.S.); e.mik@erasmusmc.nl (E.G.M.); f.harms@erasmusmc.nl (F.A.H.); 4 Department of Chemistry, Materials, and Chemical Engineering Giulio Natta, Politecnico di Milano, 20133 Milano, Italy; carmen.bax@polimi.it; 5 Department of Anesthesiology, Erasmus MC, University Medical Center Rotterdam, 3015 GD Rotterdam, The Netherlands

**Keywords:** electronic nose, exhaled breath, lipopolysaccharide, sepsis, animal model

## Abstract

Sepsis is a severe systemic condition due to an extreme response of the body to an infection. It is responsible for a significant number of deaths worldwide, and is still difficult to diagnose early. In this study, a system was developed for exhaled breath sampling in mechanically ventilated patients at the intensive care unit (ICU), together with a custom-made electronic nose (e-Nose) device for detecting sepsis in exhaled breath. The diagnostic performance of this system was evaluated in an animal sepsis model. Ten pigs (LPS group) were administered lipopolysaccharide (LPS) to induce a systemic inflammatory response. Nine other pigs received a placebo solution (control group). Exhaled breath samples were collected in Nalophan^TM^ bags and stored for temperature and humidity equilibration before e-Nose analysis. Measurements were corrected for the effects of different fractions of inspired oxygen (FiO_2_) on e-Nose sensors. Two classification models using e-Nose and physiological measurements were developed and compared. One hour after LPS administration, the e-Nose data model with FiO_2_ correction showed a higher accuracy (76.2% (95% confidence interval (CI) [58.0, 94.2])) than the physiological data model (59.0% (95% CI [39.5, 79.5])), indicating the potential of the early detection of sepsis with an e-Nose.

## 1. Introduction

Sepsis is an excessive physiological response to infection, resulting in a severe systemic condition [1]. This response can lead to organ failure or hypotension, making sepsis a leading cause of mortality in intensive care units (ICU) [2]. The annual burden of sepsis on patients and healthcare systems is significant, with 48.9 million cases and 11 million sepsis-related deaths worldwide, representing 20% of all global deaths [2]. This leads us to consider that for every 1000 hospitalized patients, an estimated 15 patients will develop sepsis as a complication of hospital admission. This emphasizes the importance of early detection and prompt intervention. Early treatment can reduce the impact of sepsis on the patient, potentially saving lives and resources. However, the early diagnosis of sepsis remains difficult [3]. Even though several biomarkers related to sepsis have been proposed, none of them have the specificity and sensitivity to be used as a single indicator of sepsis development in clinical practice [4,5,6].

Electronic noses (e-Noses) have been proven capable of diagnosing several diseases from various biological samples. E-Noses contain partially specific metal oxide semiconductor (MOS) gas sensor arrays to mimic the functioning of the mammalian olfactory system [7]. By detecting the alteration of volatile compound (VC) patterns (so-called “smell prints”) in biological fluids such as urine, feces, and breath, different types of diseases can be detected [8,9,10,11,12,13].

Despite the considerable number of scientific studies discussing the use of e-Noses as a diagnostic tool for several conditions, to the best of our knowledge, few studies have focused on the use of this technology for the detection of sepsis. Fink et al. showed that exhaled breath analysis can distinguish sepsis from inflammation in rats [14], while Kombo et al., Berkhout et al., and De Kroon et al. showed the potential of VCs in fecal samples for neonatal sepsis detection in small proof-of-concept studies [15,16,17]. Rogosh et al. [18] showed differences in exhaled breath patterns between premature neonates with and without blood stream infections, while Van der Aart et al. [19] showed 80% accuracy using a commercially available e-Nose to recognize sepsis in spontaneously breathing adult patients visiting the emergency department. The e-Nose measurements were able to identify sepsis in patients suspected of having an infection, but who were not obviously septic with an area under the receiver operating curve (AUROC) of 0.83, a sensitivity of 71%, and a specificity of 83% [19]. The lung endothelium is one of the largest substance exchange surfaces in the body, making the exhaled breath rich in endogenous VCs, reflecting the patient’s condition [20,21,22,23]. Furthermore, breath can be sampled non-invasively, making it suitable for frequent or continuous analysis to detect sepsis onset. These characteristics, along with the lack of identification of a specific sepsis-related VC, make the e-Nose a promising technology for sepsis detection.

As mechanical ventilation is often required for patients with severe sepsis and for those at risk, diagnostic methods for patients with this condition should also be applicable during mechanical ventilation. Published studies on exhaled breath analysis in mechanically ventilated subjects sampled the exhaled breath either at the endotracheal tube or in the expiratory limb [24,25,26,27]. However, these approaches dilute exhaled breath with the clean gas bias flow of the ventilator and introduce a variability of VC concentrations due to the variation in patients’ gas flow within the breathing cycle [28,29]. Moreover, sampling from the respiratory circuit may interfere with the normal operation of the mechanical ventilator control strategy, as the inspired and expired volumes measured by the device will differ due to the exhaled breath collection, which, unlike leaks, occurs only during expiration.

This paper describes an experimental setup and associated e-Nose prototype, developed for exhaled breath sampling in mechanically ventilated subjects, by integrating it in the mechanical ventilator and promoting synchronization between breathing and sampling. In addition, the experimental approach aimed at minimizing the interference of the humidity and variable Fraction of Inspired Oxygen (FiO_2_) levels on e-Nose sensor signals is described. A preliminary data analysis to evaluate the possibility of the early detection of the onset of sepsis with this device is presented.

## 2. Materials and Methods

### 2.1. Animal Population and Treatment

The developed exhaled breath collection system and e-Nose prototype were evaluated in a lipopolysaccharide (LPS) animal model. In this prospective intervention animal study, 19 female Yorkshire x Norwegian Landrace pigs (3–4 months old) were investigated at Erasmus MC, University Medical Center Rotterdam, Rotterdam, The Netherlands. Pigs were consecutively assigned to a control and LPS group in order to obtain 10 pigs in each group. During the preparation phase, pigs were sedated, anesthetized, and intubated using cuffed endotracheal tubes, and surgery was performed for invasive measurements [30]. The mechanical ventilator (Maquet Servo-i Ventilator, Getinge AB, Rastatt, Germany) was set to pressure control ventilation. Ventilation settings (breathing frequency, FiO_2_, and positive end-expiratory pressure (PEEP)) were set to maintain average end-tidal CO_2_ (etCO_2_) values at 4–6 kPa and the arterial partial pressure of oxygen between 80 and 120 mmHg.

After preparations, pigs were placed in the left recumbent position, and baseline measurements were performed. Electrocardiogram (ECG), heart rate (HR), temperature (T), oxygen saturation (SpO_2_), and etCO_2_ were continuously monitored during the entire experiment. Hemodynamics were invasively and continuously monitored using a femoral artery catheter. Sterofundin (3–30 mL/kg/h), NaCl 0.9% (2–40 mL/kg/h), and norepinephrine were continuously administered to maintain the hemodynamic state of the pig.

LPS was administered after baseline measurements with a dose of 1.75 µg/kg/h to the pigs in the sepsis group to induce systemic inflammation. After 45 min, the LPS dose was increased to 2.00 µg/kg/h, and at 90 min after baseline, to 2.25 µg/kg/h. The control group pigs received 0.9% NaCl solution in the same dose instead of LPS. Mean arterial pressure (MAP) was kept above 80 mmHg in the control group and between 40 and 60 mmHg in the LPS group. Norepinephrine administration rates were increased, and crystalloids were administered to fulfill these criteria. FiO_2_ administration was adjusted based on SpO_2_ measurements and arterial blood gas sampling.

This study was part of a study protocol for which ethical approval was received by the Central Authority for Scientific Procedures on Animals (license number AVD101002115658). The experiments were conducted according to the Dutch Experiments on Animals Act.

### 2.2. Exhaled Breath Sampling Protocol

Exhaled breath was collected in 6-L Nalophan™ bags during the experiment at eight different time points using an exhaled breath collection system. The first sample was collected at baseline before LPS or placebo administration. The other samples were collected at the following time points: 5, 30, 60, 90, 120, 150, and 180 min after initiation of LPS administration (t5–t180). For each time point, the following physiological data were recorded:

- Circulation: MAP, diastolic blood pressure (DBP), systolic blood pressure (SBP), HR, and T.
- Ventilation: FiO_2_, PEEP, and peak inspiratory pressure (PIP).

After collection, exhaled breath samples were stored for at least 12 h in the room where the e-Nose was installed to reduce the sample humidity content. High humidity levels reduce MOS sensors’ sensitivity to VC as the adsorption of water and VC on the sensor’s active layer is a competitive mechanism. The permeability of Nalophan^TM^ to water vapor allows the equilibration of internal and external moisture content within a few hours. This strategy reduces humidity interference with VC sensors’ response [31,32]. The samples were analyzed with the e-Nose within 48 h after collection to avoid sample deterioration [31].

### 2.3. Exhaled Breath Collection System

The breath collection system was designed to automatically collect exhaled breath in a repeatable manner without interfering with mechanical ventilation and to avoid external contamination from the expiration line of the mechanical ventilator.

**Figure 1:**
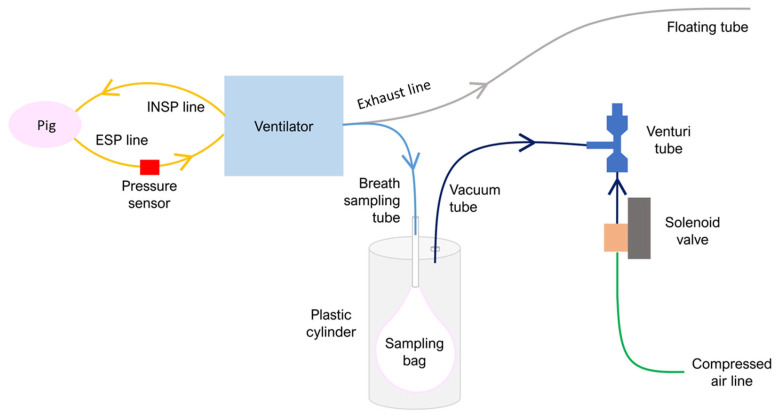
Schematic of the exhaled breath sampling system. The animal was ventilated with a two-limb mechanical ventilator fitted with an inspiratory line (INSP) and an expiratory line (ESP). A pressure sensor was placed on the expiration limb to detect the breathing phase. A long floating tube and a sampling system were connected to the exhaust line of the ventilator. The floating tube acted as an exhaled breath buffer reservoir to avoid contamination by environmental air during the sampling phase. The plastic cylinder was connected to a Venturi tube. When the pressure in the expiratory line goes below a given set value, the control unit detects the beginning of the exhalation. During exhalation, the solenoid valve opens, and the Venturi vacuum generator creates a negative pressure in the cylinder, allowing the bag to be filled with exhaled breath from the ventilator exhalation line.

The system (Figure 1) was based on an Arduino Uno (Arduino SRL, Monza, Italy) with an LCD screen and four buttons (HD44780 LCD Keypad Shield, AZDelivery, Deggendorf, Germany), allowing the user to change the settings of the system. Exhalation was identified automatically by a drop in pressure measured along the expiration line using a pressure sensor (HCLA02X5, First Sensor, Berlin, Germany). The start of exhalation was determined as a pressure decrease below a threshold set by the user according to the ventilation parameters. The end of the exhalation phase was identified as a pressure increase above the same threshold.

The collection bag was connected to one of the side-ports of a T-piece. The other two ports of the T-piece were connected to (1) the exhalation outlet of the mechanical ventilator, and (2) a 3 m long tube open to the atmosphere. This long floating tube acted as an exhaled breath reservoir to prevent ambient air from entering the sampling bag during breath collection without imposing mechanical load on the subject during expiration.

The sampling bags were inserted into a transparent plastic cylinder with an internal volume of 11 L and two 8 mm internal diameter connectors at the top. One connector was used to connect the bag to the sampling line from the T-piece. The other connector was used to apply a negative pressure inside the cylinder in order to fill the sample bag synchronously with exhalation. The negative pressure was generated by a Venturi vacuum generator (VN-10-H-T3-PQ2-VQ2-RO1 vacuum generator, Festo, Esslingen am Neckar, Germany) connected to a solenoid electronic valve (Elettropilota U2 3/2 G1/4, AB-0822, Univer, Milan, Italy), which in turn was connected to the hospital pressurized air supply. When the exhalation start was detected, the valve opened, and the air flew in the venturi tube. This generated negative pressure inside the cylinder, promoting bag inflation with the pig exhaled breath. The sampling system activated automatically with each exhalation, and this process stopped when the bag was filled. The operator can choose the number of breathing acts needed to fill the bag according to the sampling time and lung volume. All tubes in the breath collection system were made of PTFE.

### 2.4. E-Nose Prototype for Exhaled Breath Analysis

**Figure 2:**
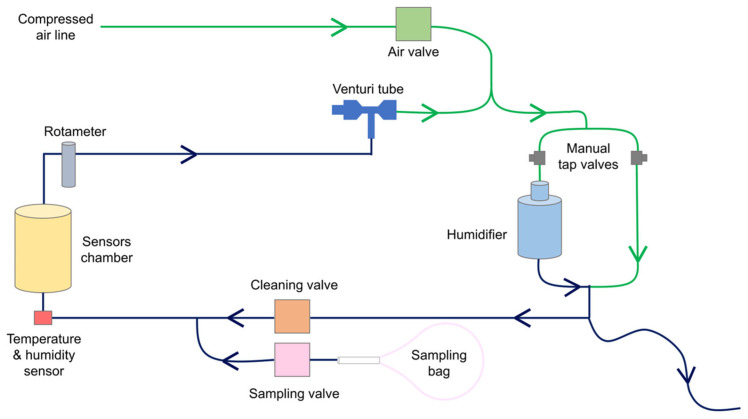
Schematic of the e-Nose analysis system developed for this study. When the system is switched on, the air valve and the cleaning valve are open, and the compressed air flows through the humidification circuit to the sensor chamber with a flow rate equal to 1 L/min. The flow rate is generated with a Venturi tube and regulated by a rotameter. This configuration is maintained during the “before” phase of the analysis. At the start of the “during” phase, the cleaning valve is closed, and the sampling valve opens. The air contained in the sampling bag flows into the sensors’ chamber. During the “after” phase, the initial configuration is restored. A long tube open to the atmosphere is connected to the humidification circuit to avoid that reference air is taken from the ambient to exit air in case of a high flow, and to prevent increased humidity in the humidification circuit during sample analysis.

The e-Nose prototype used for this study has been designed to ensure the analysis of the exhaled breath samples under repeatable and controlled flow rate, temperature, and humidity [32]. The e-Nose analysis system was connected to a compressed air line, providing clean air. The compressed air line was also connected to a Venturi tube to generate a negative pressure. The system includes the following components (Figure 2):

- A custom-made e-Nose sensors chamber containing 8 MOS sensors (TGS 2603 x2, TGS 2600 x2, TGS 2610 x1, TGS 2620 x1, TGS 2611 x1, and TGS 2602 x1, Figaro Inc., Osaka, Japan). This chamber was designed to ensure the best flow conditions for all the sensors [32,33];
- A temperature and humidity sensor (SHT40, Sensirion AG, Stäfa, Switzerland) positioned at the inlet of the sensors chamber;
- A rotameter (MR3A14BVBN, Key Instruments, Croydon, PA, USA) placed after the sensors chamber to adjust the sampling flow rate in the sensors chamber;
- A Venturi tube (VN-10-H-T3-PQ2-VQ2-RO1 vacuum generator, Festo, Esslingen am Neckar, Germany) connected to the rotameter, used as a negative pressure source to drive the sample flow into the system;
- An electro valve (2V025-08, Heschen, Foshan, China) to control compressed air entering the system;
- Two electro valves (2V025-08, Heschen, Foshan, China), used as “cleaning valve” and “sampling valve”, respectively. The cleaning valve is open before and after sample analysis to have clean air from the compressed air line flowing into the sensors’ chamber. The sampling valve opens during the sample analysis phase (called the “during” phase) to let the exhaled breath flow into the sensors’ chamber;
- A humidification circuit for humidifying the reference air and reducing the humidity difference between the sample and reference gases as the air coming from the compressed air line is dry. Water is a strong competitor of VCs in MOS surface reactions, and maintaining identical humidity levels in all experiments increases measurement reproducibility [32]. The humidification system comprises two manually operable tap valves and a water tank filled with up to 300 mL of distilled water. The compressed air was divided into two branches by adjusting the valves. The air flowing into the water tank branch was humidified and then mixed with the air coming from the second branch. The humidity of the mixed air was, therefore, set by adjusting the opening ratio of the two tap valves. A long tube open to the atmosphere was connected to the exit of the humidification circuit. The tube was used for three reasons: (1) to allow air to exit if it entered the humidification circuit with a flow higher than 1 L/min, (2) to let air flow into the humidification circuit when the cleaning valve was closed, avoiding a non-expected increase in humidity, and (3) to prevent the use of ambient air as a reference during the cleaning phase. The performance of the humidification system was evaluated by computing the humidity difference between reference air and the sample.

The e-Nose analysis system operations were managed by a microcontroller (PSoC 5LP, Infineon, Neubiberg, Germany) and a graphical user interface (GUI) developed in LabView^®^ (National Instruments, Austin, TX, USA) to activate the analysis and save the data.

The analysis of the breath samples started with an initial 45 min e-Nose cleaning period with odorless air. After this cleaning period, each measurement comprises the following phases:

1. “before” (1 min): the baseline response of the MOS gas sensors is recorded while flushing clean, humidified air into the sensors’ chamber.
2. “during” (5 min): after opening of the sampling valve, exhaled breath is drawn from the sampling bag into the sensors’ chamber, leading to the adsorption of VCs on the MOS surfaces and to a consequent change in their resistance.
3. “after” (10 min): clean air is circulated again inside the sensors’ chamber to recover to baseline conditions.

### 2.5. Classification Models

Two classification models were developed to discriminate controls from sepsis subjects. First, a classification model was built using the e-Nose data collected during the animal experiment to assess its capability in sepsis detection. Then, a second classification model was developed using only physiological parameters (MAP, DBP, SBP, HR, and T), commonly available in clinical settings. The performance of the e-Nose model was compared to that of the physiological model to evaluate the added value of the e-Nose over standard clinical monitoring. The two classification models were developed at each time point where a breath sample was collected (before the LPS/placebo administration (t0) and at t5, t30, t60, t90, t120, t150, and t180). For each time point, all the data collected at that time point were used together with the data of all previous time points since LPS administration. For example, the classification model corresponding to the sample taken at 60 min after LPS (t60) was built using the data sampled at t5, t30, and t60.

The leave-one-out (LOO) approach was implemented to obtain a test and training set, as not enough data were available to allow external validation [34]. Before training the classification models [35], the features in the training set were normalized (z-score), and Principal Component Analysis (PCA) [36] was performed to reduce dimensionality [35]. The same transformations (normalization and PCA) were applied to the test set, and classification was performed again. The accuracy, sensitivity, and specificity were calculated for each time point iteration and were presented with their 95% confidence intervals (CI). Data analysis was performed in MATLAB (version R2021b, The MathWorks, Inc., Natick, MA, USA).

#### 2.5.1. Physiological Data Classification Model

The analysis of the physiological data started with data exploration and determination of physiological parameters differentiating the control group from the LPS group. Z-score normalization [35] and PCA were applied to the data at t0. For each other time point, data were projected on the calculated PCA space. To better understand the direction of variation of each animal within the PCA space, the direction and modulus of its variation in the same space were calculated. Modulus and direction were then multiplied, and the mean for each group was calculated.

Five components of PCA were calculated, and a logistic regression classification model was trained. Logistic regression was chosen as it is easy to interpret and is very efficient to train [36].

#### 2.5.2. E-Nose Data Classification Model

##### FiO_2_ Correction

Before the classification model using e-Nose data was developed, an algorithm for FiO_2_ correction was created. During the experiments, FiO_2_ administration to the pigs was adjusted based on SpO_2_ measurements and arterial blood gas sampling. This introduced further variability into the e-Nose measurements because the sample oxygen concentration affects the MOS sensors’ responses. N-type MOS sensors were expected to increase their resistance in the presence of oxygen [37]. To correctly interpret our results obtained during different FiO_2_ values, a specific FiO_2_ compensation algorithm was introduced. This algorithm corrected for FiO_2_, in a similar method as the MOS sensors’ response correction performed when operating with variable humidity levels [38,39].

To develop the compensation algorithm, it was necessary to first characterize the effect of FiO_2_ on the MOS responses [38,39]. We repeated a sequence of tests with a mechanical test lung connected to the ventilator and set different FiO_2_ values (21%, 25%, 30%, 35%, 40%, and 50%). The FiO_2_ levels were chosen to be similar to those used during the experiments.

Three samples were collected for each FiO_2_ level on two subsequent days. On day 1, two bags were filled in sequence for each FiO_2_ level_._ Ventilator parameters were kept stable for each FiO_2_ value. The third bag series was sampled the next day after switching off the ventilator overnight.

After storing the bags for 12 h, the samples were analyzed by the e-Nose in random order. The oxygen concentration in the bags was measured with a commercial oxygen sensor (Gasboard-2510, Cubic, Wuhan, China) before and after the 12 h storage to verify that the oxygen does not diffuse through the Nalophan™.

For each MOS sensor, the mean of the three sensor response curves was calculated for each FiO_2_ level. Two different correction strategies were considered for each sensor response, as summarized in Table 1. *R_bas_* is the sensor response to be corrected, and *R_s_* is the resistance of the sensor during tests at the different FiO_2_ values. Each correction function was a bivariate function (time and FiO_2_ dependent).

**Figure 3:**
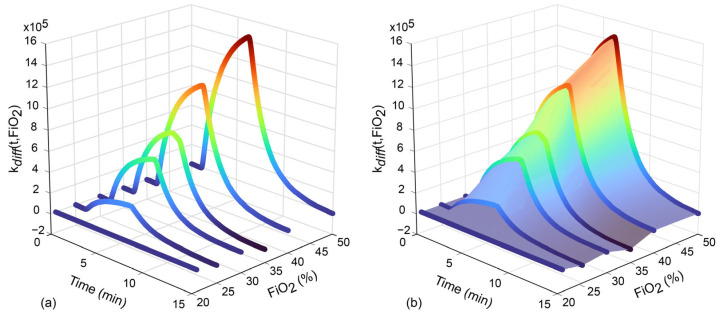
Example of correction function *k_diff_*(*t*,*FiO*_2_) for MOS sensor TGS 2603. (**a**) All e-Nose response curves at different FiO_2_ levels (21%, 25%, 30%, 35%, 40%, and 50%) over time and the point-by-point difference between the curve at FiO_2_ of 21% and the other FiO_2_ curves. (**b**) An example of the linear interpolating surface for this curve. *MOS*: metal oxide semiconductor; FiO_2_: fraction of inspired oxygen.

With the first strategy (Figure 3), the point-by-point difference between the curve obtained at FiO_2_ = 21% and all other FiO_2_ levels was computed. The correction function *k_diff_*(*t*,*FiO*_2_) was defined as the linear interpolant of this difference, resulting in a dependence on time and FiO_2_ level. To apply the correction to the pig data, the baseline was removed from the whole response curve of the pigs (*R_s_* − *R_s_*(t = 0), where *R_s_*(t = 0) was the sensor’s resistance baseline). The *k_diff_*(*t*,*FiO*_2_) value corresponding to the FiO_2_ of the analyzed sample was subtracted from the baseline-corrected response curve of the pig data.

With the second strategy, the point-by-point ratio (instead of difference) between the curve obtained at FiO_2_ = 21% and the other FiO_2_ levels was computed. The correction function *k_ratio_*(*t*,*FiO*_2_) was defined as the linear interpolant of the ratio, resulting in a dependence on time and FiO_2_ level. To apply this correction to the breath samples of the pigs, the baseline was first corrected by dividing the whole curve by the curve at baseline. The *k_ratio_*(*t*,*FiO*_2_) corresponding to the FiO_2_ of the analyzed sample was computed, and each point of the baseline-corrected response curve was divided by the corrected function.

##### Classification

After signal correction with the two proposed strategies, curves were visually analyzed, and features were extracted from the curve to characterize the shape and the dynamics of the resistance time courses. Examples of features are the response amplitude, the area under the curve, and the curve slope [40]. These features were used to build the classification model. Outliers were determined for each time point and feature, using the interquartile range (IQR) [33]. If a feature value was outside the IQR, this outlier was eliminated. PCA was run on two components, and a decision tree was chosen as a classification model for its simplicity and ease of use [41].

## 3. Results

### 3.1. Exhaled Breath Collection System

The newly developed sampling system allowed the automatic sampling of exhaled breath during all experiments. It was easy to use, and no impact on the mechanical ventilation or the animals’ conditions was reported. The FiO_2_ measured at the beginning and at the end of the storage in bags did not change, confirming that storage in Nalophan™ did not cause any O_2_ loss.

### 3.2. E-Nose Prototype for Exhaled Breath Analysis

No issue related to the e-Nose analysis system was reported during this study, neither in malfunctioning components nor in data loss due to system issues. The integrated humidification system in the e-Nose setup completed its goal of maintaining the constant humidity of the reference air. It reduced the difference between the reference air and the target gas humidity to a median absolute humidity difference of 5.1 ± 0.1 g/m^3^ along the experiments.

### 3.3. Animal Experiments

Exhaled breath samples were successfully collected and analyzed in 9 control pigs and 10 pigs administered LPS. Data from one control pig were excluded due to corruption caused by a saving error. Two pigs were a priori excluded from the analysis and replaced to obtain ten pigs in each group. One pig did not meet the assigned group criteria and the other pig died prematurely. One pig from the control group was post hoc excluded (and not replaced) as the baseline bag was not correctly collected and was, therefore, unreliable. The pigs had a weight of 29.9 ± 2.4 kg (mean ± standard deviation), which did not differ between groups.

### 3.4. Physiological Data Classification Model

**Figure 4:**
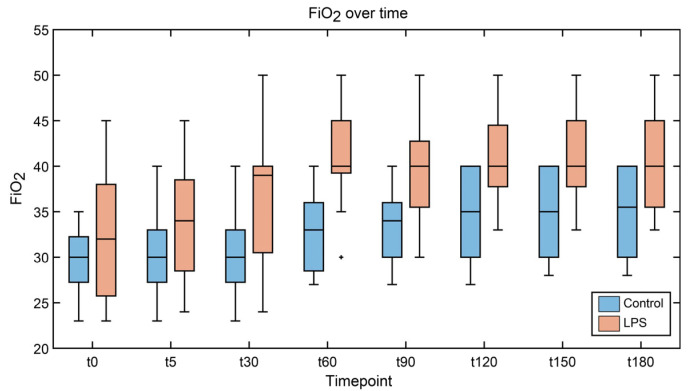
Median and interquartile range (IQR) of FiO_2_ in the ventilation settings for pigs in the control (blue) and LPS (orange) groups. FiO_2_: fractions of inspired oxygen; LPS: lipopolysaccharide.

Figure 4 reports the FiO_2_ variation relevant to the different groups (i.e., controls vs. LPS subjects). As the initial value of FiO_2_ was different among the experiments due to the different baseline conditions of the animals, the increase in FiO_2_ after the administration of LPS or the placebo solution is reported. In particular, 1 h after LPS administration, the FiO_2_ difference was larger in the LPS group compared to the controls. FiO_2_ is a key element in this study because an increase in FiO_2_ indicates clinical deterioration. After all, the oxygen concentration of the samples affects the sensors’ response, as discussed in Section 2.5.2.

**Figure 5:**
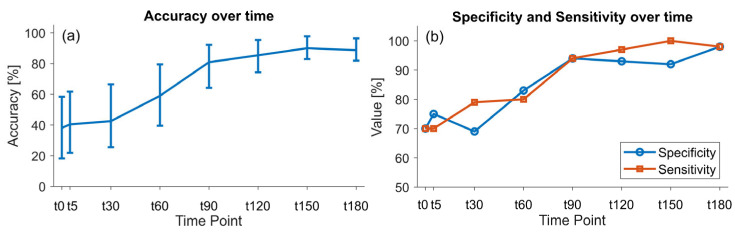
Performance over time of the classification model using physiological data. The model classified e-Nose data into a control and LPS group. (**a**) Accuracy. (**b**) Sensitivity and Specificity. LPS: lipopolysaccharide.

The classification model using physiological data showed a maximal accuracy of 90.0% (95% CI [82.3, 97.7]) in discriminating controls from septic subjects at t180 (Figure 5a). Figure 5b reports that the sensitivity and specificity increase over time.

### 3.5. E-Nose Data Classification Model

**Figure 6:**
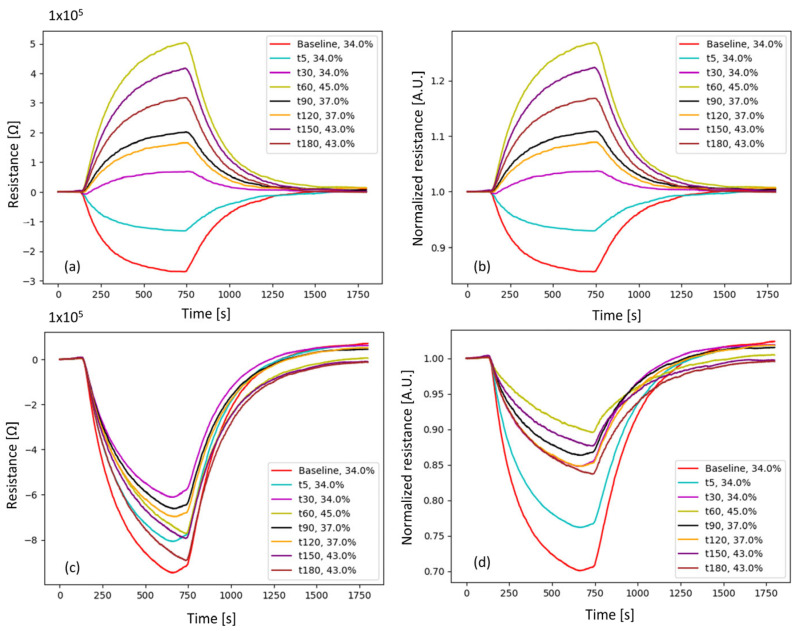
Example of the response curve of MOS sensor TGS2603 for different experimental time points. Each time point has different FiO_2_ settings. The curves are shown without (upper panels) and with corrections (lower panels). (**a**) Original sensor response with baseline removed (*R_s_* − *R_s_*(t = 0)). (**b**) Original sensor response with baseline corrected (*R_s_*/*R_s_*(t = 0)). (**c**) Resulting response after the application of *k_diff_*(*t*,*FiO*_2_) correction. (**d**) Resulting response after *k_ratio_*(*t*,*FiO*_2_) correction. The agenda reports the time point and corresponding FiO_2_ values. A.U. = arbitrary units; FiO_2_: fraction of inspired oxygen.

In Figure 6, representative response curves of the MOS sensors at different time points show the effect of an FiO_2_ increase on the sensors’ responses. Figure 6a,b show a change in the direction of the response: when FiO_2_ increases, the resistance increases. In Figure 6c,d, the resulting response curves after the proposed corrections are reported. All corrected e-Nose responses showed a reduced resistance when exposed to exhaled breath. Although less evident than in the non-corrected data, the differences in response due to higher FiO_2_ values are visible.

**Figure 7:**
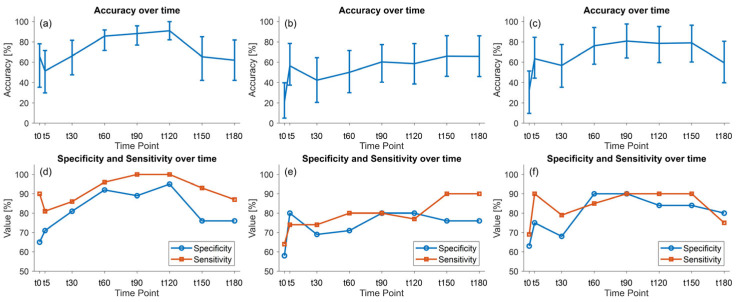
Performance of the leave-one-out (LOO) cross validation of the classification model using the e-Nose data. Accuracy with confidence intervals (**top**) and misclassification rates (**bottom**) are shown using raw e-Nose response data as inputs of the model (**a**,**d**); e-Nose response data corrected per FiO_2_ with the *k_diff_*(*t*,*FiO*_2_) correction (**b**,**e**); and e-Nose response data corrected per FiO_2_ with the *k_ratio_*(*t*,*Fi*O_2_) correction (**c**,**f**). *k_diff_*(*t*,*FiO*_2_): correction function; FiO_2_: fraction of inspired oxygen.

In Figure 7, the accuracy, sensitivity, and specificity resulting from the classification model using e-Nose data are reported both with and without the FiO_2_ correction. The classification accuracy of the e-Nose data without correction for FiO_2_ reached up to 91% (95% CI [82.1, 99.9]) at t120 and then dropped back to 62% (95% CI [42.1, 81.9]) at t180. The models using the correction functions showed a lower classification performance: the *k_diff_*(*t*,*FiO*_2_) correction showed a maximum accuracy at t150 66.0% (95% CI [46.1, 86.1]), whereas with the *k_ratio_*(*t*,*FiO*_2_) correction, a maximum accuracy of 80.9% (95% CI [64.2, 97.6]) was achieved at t90. Performance metrics of the various developed models at t60 are reported in Table 2 as an example. When comparing the performance of the two correction models, the classification with *k_ratio_*(*t*,*FiO*_2_)-corrected data showed the best performance (76.2 (95% CI [58.0, 94.2])) for detecting sepsis at this time point.

## 4. Discussion

This study reports a system for exhaled breath collection and analysis using an e-Nose during mechanical ventilation and shows preliminary data on the performance of this system for detecting sepsis onset in pigs. The sampling system is effective in collecting exhaled breath during mechanical ventilation using synchronized sampling from the ventilator outlet. This system operated without requiring special connections to the mechanical ventilator or interfering with its operations. Furthermore, this study shows effective experimental procedures and algorithms developed to mitigate the effects of confounding factors for MOS sensors, i.e., FiO_2_ and humidity. Lastly, a preliminary data analysis demonstrated, despite the limited data set, the potential of e-Nose measurements for the early detection of sepsis.

Despite the increasing number of e-Nose applications in breath analysis and the potentialities of such a technology for monitoring patients with suspected sepsis or at risk of developing sepsis, to the best of our knowledge, only one recent study addressed this application in spontaneously breathing adult patients, showing highly promising results [19]. Similarly, there is a limited number of studies using e-Noses in mechanically ventilated patients, a fragile population at high risk of developing sepsis and who would benefit from early diagnosis and treatment. This is most probably due to the lack of standardized methods for breath sampling and analysis in mechanically ventilated patients. Previous studies in the field collected exhaled breath from the expiratory limb of the respiratory circuit, i.e., between the patient and the mechanical ventilator [24,25,26,27]. Even if potentially effective, the previous approach can interfere with the mechanical ventilator control system. Gas sampling from the patient’s circuit will create a difference between the insufflated volume and the volume measured at the exhalation port. They consider this difference as a leak and manage it with the assumption that this leak flow depends only on the circuit pressure and occurs during both inspiration and expiration. However, this is not the case for exhaled gas analysis sampling systems, which only sample during exhalation. This violation of the assumption could lead to unpredictable responses and may impact the leak detection and compensation algorithms based on the assumption.

The exhaled breath sampling system developed in this study proved suitable for use in an ICU, as it was effective in collecting exhaled breath samples without interfering with standard clinical procedures. Possible improvements could focus on reducing the effort needed by the clinicians to provide an adequate sample, increasing the automatization of data collection, and setting the different parameters. These improvements will enable the use of this approach both during spontaneous breathing and mechanical ventilation. As sepsis often develops before ICU admission and mechanical ventilation is often part of the therapy, a sepsis onset detection method should be operable during spontaneous breathing [3]. Monitoring in mechanically ventilated subjects by exhaled breath analysis could help the early detection of the development of new infections (like for ventilator-associated pneumonia [42]) or, in general, detect patient deterioration.

One of the innovative features of our approach is the humidification circuit used to humidify the reference air. This humidification circuit allows the use of compressed air as a stable and clean reference gas. The use of this system had two aims. At first, it increased the humidity of the compressed air, thereby reducing the difference in the water content between the reference air and the breath sample. The second aim was to maintain this difference constant during the whole measurement.

The main limitation of the proposed hardware is that it does not provide real-time analyses. In order to reduce the sample humidity, the bag can only be analyzed several hours after the collection, making the result availability late. Future work will be focused on improving the time required by the methods for humidity management and data analysis, which are now the most relevant limiting factors for the implementation of this technology for clinical applications [32].

A noteworthy limitation of the applied animal model relates to the discussion regarding the applicability of LPS models for sepsis research. The LPS model is an endotoxin model inducing a subset of the physiological changes that occur with sepsis [43,44]. However, the fact that measurable differences in VCs occurred between the control group and LPS group, suggests that in clinical sepsis, these differences might be more evident as a broader pallet of VCs might be present. Clinical studies could provide more clarity on this hypothesis.

A preliminary data analysis was performed to evaluate the performance of the e-Nose in detecting the onset of sepsis in an animal model. The variability of the sensors’ response by the variation of FiO_2_ during the measurements required FiO_2_ correction. Two linear correction models were considered: the first correction used point-by-point subtraction, the second, the point-by-point ratio. Both models were built with clean air samples collected and analyzed using the same procedure for exhaled breath data. This data correction was not only important to reduce sensor variability, but also because the physiological status of the animal and FiO_2_ are highly correlated [45]. This could lead to performance bias.

Classification models were built and trained, and accuracy was calculated for each time point. Both the classification performed on physiological data and the one performed on non-corrected data reached a high level of accuracy. Despite these results, it is important to remember that the considered sensors’ data were highly influenced by the oxygen content, which was related to the severity of the patient’s condition. Further studies should clarify the impact of different FiO_2_ levels on the MOS sensors’ response to develop more sophisticated correction models based on the obtained knowledge on the underlying mechanism.

At t60, one hour after the LPS administration, the e-Nose data with *k_ratio_*(*t*,*FiO*_2_) correction showed higher accuracy than the physiological data, suggesting that the e-Nose can distinguish the onset of sepsis early with adequate data correction. At later timepoints, the physiological data model outperformed the sepsis detection performance of the e-Nose models. To increase the level of evidence of this preliminary study, future studies with larger sample sizes should be performed and more advanced data classification models implemented.

## 5. Conclusions

Sepsis is still a serious, life-threatening condition associated with high mortality rates. Novel sensitive, specific, and clinically applicable tools are warranted to start sepsis treatment early, as early symptoms are subtle and non-specific. This paper presents a novel setup for exhaled breath sampling and analysis using an e-Nose during mechanical ventilation for the early detection of sepsis. The performance was evaluated in an animal model. The sampling and e-Nose analysis prototypes proved feasible without interfering with the standard clinical procedures or mechanical ventilation. Further studies should focus on the automatization of the sampling and analysis processes, and on the real-time acquisition of results. Preliminary data analysis suggests that the approach may detect sepsis onset earlier than physiological data; further investigations with a larger sample size are required to increase the level of evidence.

## Figures and Tables

**Table 1:** FiO_2_ correction strategies.

	Strategy 1: *k_diff_*(*t*,*FiO*_2_) Correction	Strategy 2: *k_ratio_*(*t*,*FiO*_2_) Correction
Mean MOS sensor response curve at specific FiO_2_ series	$R_{F_{mean}}\left(t,FiO_{2}\right)=\frac{\sum_{i}^{3}{R_{S}}_{\#i}(t,\;FiO_{2})}{3}$	$R_{F_{mean}}\left(t,FiO_{2}\right)=\frac{\sum_{i}^{3}{R_{S}}_{\#i}(t,FiO_{2})}{3}$
Bivariate correction function $k\left(t,FiO_{2}\right)$	${k_{diff}(t,FiO_{2})=R}_{F_{mean}}\left(t,FiO_{2}\right)-R_{F_{mean}}\left(t,FiO_{2}=21\%\right)$	$k_{ratio}(t,FiO_{2})=\frac{R_{F_{mean}}\left(t,FiO_{2}\right)}{R_{F_{mean}}\left(t,FiO_{2}=21\%\right)}$
Samples’ curves after baseline removal	$R_{bas}\left(t\right)=R_{S}\left(t\right)-R_{S}(t=0)$	$R_{bas}\left(t\right)=\frac{R_{S}\left(t\right)}{R_{S}(t=0)}$
FiO_2_-corrected samples’ curves	$R{\left(t,FiO_{2}\right)}_{corr}=R_{bas}\left(t,FiO_{2}\right)-k_{diff}(t,FiO_{2})$	$R{\left(t,FiO_{2}\right)}_{corr}=\frac{R_{bas}\left(t,FiO_{2}\right)}{k_{diff}(t,FiO_{2})}$

**Table 2:** Performances of the developed models at t60.

	Accuracy (%)	95% Confidence Interval
Physiological data	59.0	[39.5, 79.5]
Raw e-Nose data	85.7	[71.6, 91.8]
e-Nose data with *k_diff_*(*t*,*FiO*_2_) correction	48.1	[27.0, 69.2]
e-Nose data with *k_ratio_*(*t*,*FiO*_2_) correction	76.2	[58.0, 94.2]

Data are presented as median with 95% confidence intervals.

## Data Availability

Data are available upon request due to ethical reasons.
